# Development of a Technique Using Artificial Membrane for In Vitro Rearing of Body Lice *Pediculus humanus humanus*

**DOI:** 10.3390/insects15030145

**Published:** 2024-02-21

**Authors:** Alissa Hammoud, Meriem Louni, Linda Abou-Chacra, Gabriel Haddad, Noelle Mazzotti, Florence Fenollar, Oleg Mediannikov

**Affiliations:** 1 IHU Méditerranée Infection, 13005 Marseille, France; alissa.h.hammoud@gmail.com (A.H.); louni_meriem@yahoo.fr (M.L.); gabrielhaddad92@gmail.com (G.H.);; 2 Aix-Marseille University, IRD, APHM, MEPHI, 13005 Marseille, France; 3 Aix Marseille University, IRD, APHM, VITROME, 13005 Marseille, France; 4 Faculté des Sciences Médicales et Paramédicales, Timone, Aix Marseille University, 13005 Marseille, France

**Keywords:** body lice, vectors, in vitro rearing, in vivo rearing, Hemotek, Petri dish, scanning electron microscopy (SEM)

## Abstract

**Simple Summary:**

Louse-borne diseases have caused millions of deaths around the world and are currently re-emerging in some countries. Implementing a simple and accessible lice-rearing system would significantly advance research on the louse–pathogen cycle and vectorial capacities. Several in vivo and in vitro lice-rearing methods have been developed. However, these approaches have drawbacks, making lice production more difficult. Here, we aimed to adapt the Orlando (Or) strain of body lice on an artificial membrane. The Hemotek system and a Petri dish system covered with a Parafilm membrane were tested on newly hatched first-stage larvae (L1). Rearing follow-up consisted of recording dead, fed and moulted specimens throughout the experiments. In addition, microscopic ultra-structures, blood meal digestion and sterility were evaluated and compared to those of larvae being reared on rabbit hosts. When using heparinised blood on a Petri dish, we were able to maintain one generation of body lice. Development into adulthood was recorded 21 days after hatching, and 52 eggs were deposited. Inspection of the blood meal revealed a colour difference among lice fed in vitro and in vivo, while microscopic investigations did not show any differences. The in vitro lice-rearing experiments were conducted in accordance with animal welfare requirements and therefore have the potential to replace animal models in various biological assays.

**Abstract:**

Human lice are the only hematophagous ectoparasites specific to human hosts. They transmit epidemic typhus, trench fever and relapsing fever, diseases which have already caused millions of deaths worldwide. In order to further investigate lice vectorial capacities, laboratory-controlled live lice colonies are essential. Previously developed lice-rearing methods significantly advanced research on louse-borne diseases and louse biology. In this study, we aimed to develop a rearing technique for the Orlando (Or) strain of body lice on an artificial membrane. We tested two systems, namely the Hemotek feeding system and a Petri dish with the lice being fed through a Parafilm membrane. Lice longevity and development were drastically affected by the blood anticoagulant. Additionally, heparinised human blood on a Petri dish was the best candidate when compared to the control group (reared on a rabbit). Therefore, this strategy was applied to 500 lice. Development into adulthood was recorded after 21 days (17 days for the rabbits), and 52 eggs were deposited (240 for the rabbits). In this study, we were able to maintain one generation of body lice on an artificial membrane with comparable feeding and longevity rates to those fed on live rabbits. However, lice fecundity decreased on the artificial membrane. In vitro lice-rearing experiments will enable pathogen infection assays and pesticide bioassays to be carried out in accordance with animal welfare requirements.

## 1. Introduction

The human louse, *Pediculus humanus*, is an obligate ectoparasite that comprises two ecotypes, the head and body louse; each ecotype has its distinct ecology. The body louse, *Pediculus humanus humanus*, is a major vector of bacterial pathogens that cause severe diseases, including epidemic typhus, relapsing fever and trench fever [1], and is also implicated in the propagation of the plague pandemic [2].

The control of these re-emerging diseases is based on effective louse-control strategies, which have been proven unsuccessful until now. Therefore, novel approaches for lice control as well as new insecticides are required [3]. The difficulty of producing and sustaining human lice colonies in a laboratory setting limits fundamental research into human lice biology, vector–pathogen interactions and the development of control methods against these insects. Researchers have experimented and developed several in vivo and in vitro rearing systems. However, it has always been challenging to overcome the difficulties associated with the laboratory rearing of lice [4].

In natural conditions, both head and body lice have constant access to their host and consume several daily blood meals [5]. Indeed, it is the principal limiting factor in establishing human lice colonies in vitro. In the laboratory, reproducing the same biological phenomenon continues to be problematic and difficult [4]. For many years, human lice rearing was practiced on human volunteers using different methods. Mainly, individuals wore a capsule containing living lice, strapped directly to their body, mostly all day long, allowing the lice to blood-feed freely through a wired mesh [6]. This approach is still used nowadays, despite the negative repercussions for the volunteers [7].

Over time, the body louse has adapted to feeding on rabbits, the only substitute host known so far [8,9]. Rabbits are commonly used to maintain body lice colonies, to perform immunological and toxicological studies, as well as experimental infections with louse-borne pathogens [10,11,12,13,14,15,16,17,18]. This method is conditional upon approval from the Institutional Animal Care and Use Committee, and requires trained staff in addition to burdensome manipulations of vertebrate animals. The rabbits must be anaesthetised and laid on their backs with their forelegs and hind legs attached. The body lice are only allowed to suck blood on the rabbit’s shaved abdomen [9]. Despite the fact that this feeding method is effective in maintaining lice rearing, it is barely compatible with animal welfare principles. In addition, the cost of accessing and maintaining vertebrate animals may be impossible for some laboratories [4]. Therefore, establishing in vitro feeding techniques for rearing lice is indispensable.

Several in vitro approaches for body lice feeding have been developed. Among them is one that consists of warming animal blood in different devices covered with a membrane. Blood is usually warmed by a hot plate or a circulating water bath [19,20,21,22]. A recent technique (2020) was reported using the Hemotek apparatus, a commercially available blood-feeding device which has been successfully used to feed mosquitos [4,23,24]. Using these approaches, the results demonstrate that in most cases, low feeding or high mortality rates occur, making them insufficient to support the lice development [19,21]. However, lice feeding adaptation had also been reported using several automated and manual in vitro rearing systems that enable successful head and/or body lice colony propagation [7,25,26]. Until now, these remain the only described options for rearing human lice without the use of animals or human hosts.

Based on the data found in the literature, we aimed to develop a rearing technique, starting by evaluating the efficiency of different lice-rearing methods, as well as maintaining body lice colonies on an artificial membrane, in order to enable the study of lice morphology and biology, vector-borne pathogen interactions and insecticide tolerance.

## 2. Materials and Methods

### 2.1. Body Louse Strain and Conservation Condition

Since 1946, the Orlando body louse strain (Or) has been maintained on New Zeeland female rabbits (Charles River Laboratories, St Germain sur l’Arbresle, France) [9]. In our laboratory, *P. h. humanus* rearing was initiated in 1995. Lice were maintained in ventilated plastic jars on a small piece of black cotton cloth, in favourable conditions, at 29–30 °C and 70–80% of relative humidity (RH). Newly hatched unfed first-stage larvae (L1) from the Or strain were used in each of the tests, and feeding was performed every 48 h.

### 2.2. In Vitro Louse Maintenance

#### 2.2.1. Artificial Blood-Feeding Apparatus: Rearing System

**Figure 1:**
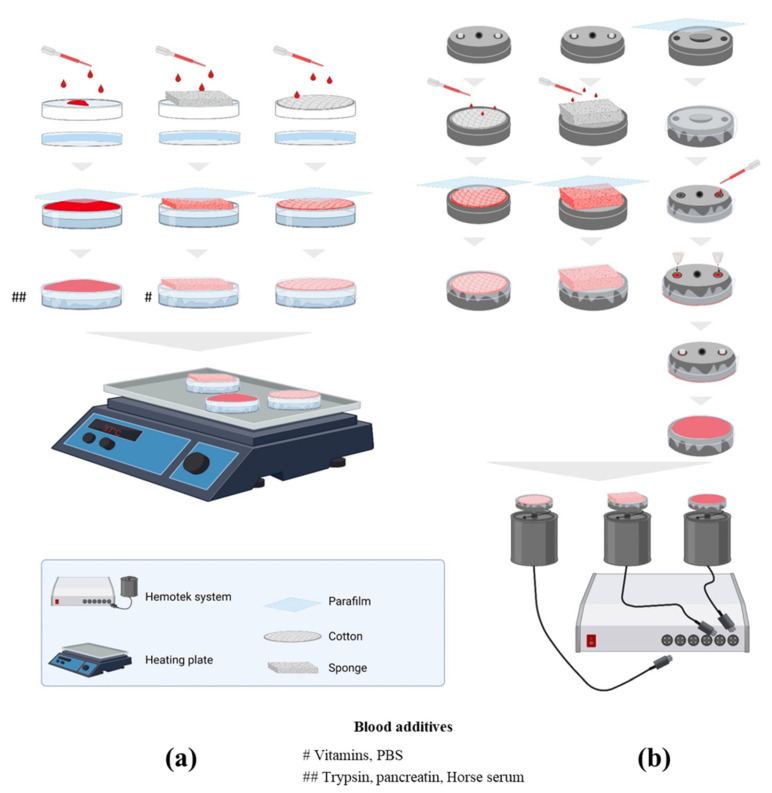
Strategy of the preparation protocol of the used lice-rearing systems. (**a**) Blood meal preparation on Petri dish using different blood supports and blood additives. (**b**) Hemotek system feeder preparation. Citrated and heparinised blood were offered as blood meals in both systems.

Two artificial-membrane feeding systems were used. First, a Hemotek device (Hemotek Ltd., Blackburn, UK, http://hemotek.co.uk/products, accessed on 1 March 2022) was implemented following the manufacturer instructions. Metallic blood reservoirs (product code: OR37-25) were disinfected by soaking in a 10% bleach solution for 30 min, and then, rinsed with sterile water and dried before and after use. For the blood meal preparation, a Parafilm membrane was stretched over the blood reservoir and 2 mL of human blood was added; the reservoir was subsequently plugged with plastic stoppers (Hemotek product code PP5-250). Finally, the prepared reservoir was screwed into an FU1 feeder unit (Hemotek product code FU1-0) previously heated to 37 °C. The second feeding system consisted of a sterile Petri dish deposited on a hotplate. Briefly, the inside of the Petri dish was filled with water, and afterwards, the lid was placed and 2 mL of human blood was added on top of the Petri dish lid. A Parafilm membrane was stretched over the lid, trapping the blood meal between the lid and the Parafilm membrane. The Petri dish holding the blood meal was set up on a hot plate and maintained at 37 °C (Figure 1). All these procedures were carried out under a level 2 biosafety hood.

#### 2.2.2. Blood Meal Support

For each rearing system, different blood meal supports, such as cotton discs (1) and kitchen cleaning sponge wipes (2), were considered. Cotton discs purchased from a nearby shop (Cien Cotton Wool Soft Makeup Remove Pads, Lidl, 18 Boulevard Jean Moulin, 13005 Marseille, France) were soaked with blood, deposited inside the metallic blood reservoir (Hemotek system) or on top of the Petri dish lid (Petri dish system) and covered with a stretched Parafilm membrane. The sponge was also purchased from a nearby shop (Spontex 4 Carréponge Super Absorbant et Anti-Odeur, Casino Supermarché, 22 Boulevard Jean Moulin, 13005 Marseille, France), washed with sterile water and natural soap (Savon Codex, Savon Liquide Glycériné. Laboratoire du Solvirex, 92120 Montrouge, France) for disinfection, and then, soaked in PBS 1X solution (Gibco, Life Technologies, Bleiswijk, The Netherlands) overnight before being cut into 2 cm squares and dried under a biosafety hood. The sponge squares were imbibed in blood and covered with a Parafilm membrane as previously described (Figure 1). Any trapped air bubbles were removed by gently pressing on the Parafilm membrane.

#### 2.2.3. Blood Meal Composition

The blood meal offered to the lice consisted of whole human blood mixed with two types of anticoagulants. Citrated A+ blood and mostly O+ heparinised blood units were provided by the Établissement Français du Sang (EFS), distributed into 15 mL sterile tubes under a level 2 biosafety hood, and stored at 4 °C for later use. In order to improve the blood meal quality, vitamins, PBS, trypsin and pancreatin were added to the blood meal. Seven essential vitamins for louse development (namely thiamine (B1), riboflavin (B2), niacin (B3), pantothenic acid (B5), pyridoxine (B6), biotin (B7) and folate (B9) (Sigma-Aldrich, Saint-Quentin Fallavier, France)) were added at the same concentration as in the insect cell culture medium, Dulbecco’s Modified Eagle’s Medium (DMEM). PBS solution (2.5%) was also added to the heparin-sponge Petri dish trial in order to dilute the blood meal. Finally, the effects of trypsin and pancreatin (Sigma-Aldrich, Saint-Quentin Fallavier, France) on lice longevity were tested at 0.05% on the heparin Petri dish system (Figure 1). We also applied urea and artificial sebum on the membrane to attract lice to pierce it.

### 2.3. In Vivo Louse Maintenance

Control group was reared on rabbits in this study. New Zealand female rabbits were anaesthetised with an intramuscular injection of a combination of xylazine and ketamine (35 mg/kg and 5 mg/kg). The unresponsive animal was laid on its back and had its abdomen shaved; then, the lice were fed on it for 30–40 min. The fed lice were removed onto a small piece of black cotton cloth and maintained in favourable conditions at 29–30 °C and 70–80% of relative humidity.

### 2.4. Rearing Follow-Up

#### 2.4.1. Surveillance

For each condition, a follow-up sheet was generated including the date of the experiment, the development stage of lice and the number of live, dead and engorged specimens. For each meal, the blood group, donation date, number of produced eggs and exuviae were noted. During feeding, lice were observed using a binocular magnifier to visualise the activity of the specimen. Lice from all development stages were removed for microscopic examination. In addition, during each feed, the engorgement rate was assessed by counting the number of lice that acquired their blood meal (remarkable growth in body size).

#### 2.4.2. Electron Microscopy

A follow-up using scanning electron microscopy was performed on lice from different stages throughout the rearing. Louse preparation consisted of serial dehydration for two minutes in 30%, 50%, 70%, 90% and 100% ethanol, followed by immersion for five minutes in HDMS solution. After drying, the specimens were fixed on double-sided carbon tape and observed on a scanning electron microscope (SEM) (TM 4000Plus, Hitachi, Tokyo, Japan) using the back-scattered electron detector with a 10 kV electron beam, and magnifications ranging from ×30 to ×3000.

#### 2.4.3. Blood Meal

Digestion of the blood meal was evaluated by freezing live specimens 24 h after feeding. Frozen lice were then crushed between two glass slides to extract the louse blood meal, which was then observed using optical microscopy (Zeiss Axio Zoom.V16, Zeiss, Marly le Roi, France). Furthermore, the presence of contamination in the attributed blood meal was investigated by spreading the blood meal on 5% sheep-blood-enriched Columbia agar plates (COS).

### 2.5. Strategy Validation

After carrying out the initial steps of the experiment, which included testing different feeding systems, anticoagulants and supports on 50 L1, the condition that yielded the best results was selected. This condition was then applied to 500 L1 specimens in duplicate. These results were compared to those of rearing on live rabbit hosts.

### 2.6. Cost Calculations and Statistical Analysis

It was expected that the cost of lice reared on live hosts would be higher than for those reared on an artificial membrane. We aimed to investigate this hypothesis further by calculating the cost of each experiment. The items that have been bought only once since the installation of the animal unit were considered to have a lifespan of 15 years (items such as the incubator, vacuum cleaner, cages, fridge, technician training, water bath, heating plate and Hemotek system).

To compare the number of live body lice according to each specific time, rearing system, blood types and supports, we used multivariable Poisson regressions. Data were compared to the control group live rabbits (rab). In addition, data were compared to the best result (BR). A two-sided α value of less than 0.05 was considered to be statistically significant. Statistical analyses were performed using SPSS software version 26.0 (IBM, Armonk, NY, USA).

## 3. Results

### 3.1. General Observation and Electron Microscopy

**Figure 2:**
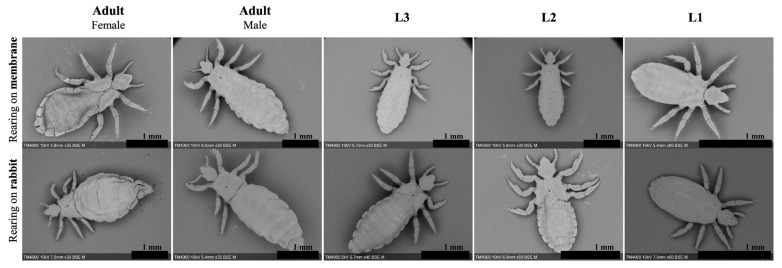
SEM micrographs showing full dorsal view of adult female, male and prior development stages of lice reared on Petri dish with heparinised blood compared to a rabbit host. Scale bar shows 1 mm.

**Figure 3:**
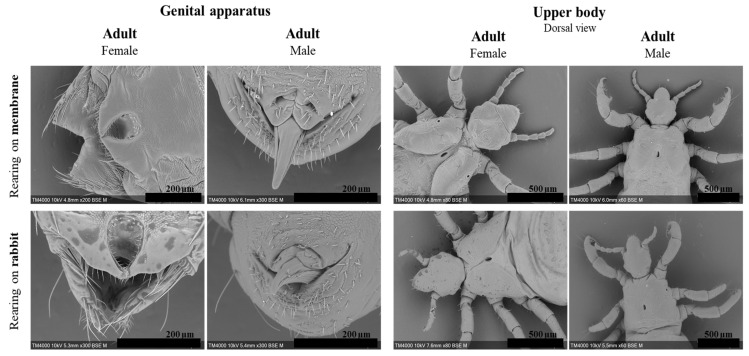
SEM micrographs of the genital apparatus and thorax ultra-structural details comparing adult females and males reared artificially and on a rabbit host.

Lice fed on rabbits lived an average of 35 days with 50% mortality on the 15th day. First-stage larvae (L1) moulted on day 7; moreover, the development into second-stage larvae (L2) was marked by segment formation and the appearance of a pore between the louse’s dorsal thorax plates (Figure 2, L1 1.34 mm, L2 1.69 mm). Eleven days post-hatching, L2 considerably grew in size and developed into third-stage larvae (L3) (2.35 mm). Differentiation into adult females (3.15 mm) or males (3.14 mm) occurred on the 17th day post-hatching (Table 1 and Figure 2). Lice that developed into adults produced 275 eggs overall with an average of 1.9 eggs per female per 48 h and an average hatching rate of 78.5% (Table 2). Adult females continued ovipositioning for 17 days with a two-day pre-ovipositional period. Furthermore, SEM micrographs identified morphological differences between male and female lice. Female lice were wider (1.40 mm for females vs. 1.01 mm for males) with a more defined thorax anatomy (Figure 3). There were no obvious differences in the mouthparts of lice in different development stages (Figure S1).

### 3.2. Blood Meal

#### 3.2.1. Anticoagulants

**Figure 4:**
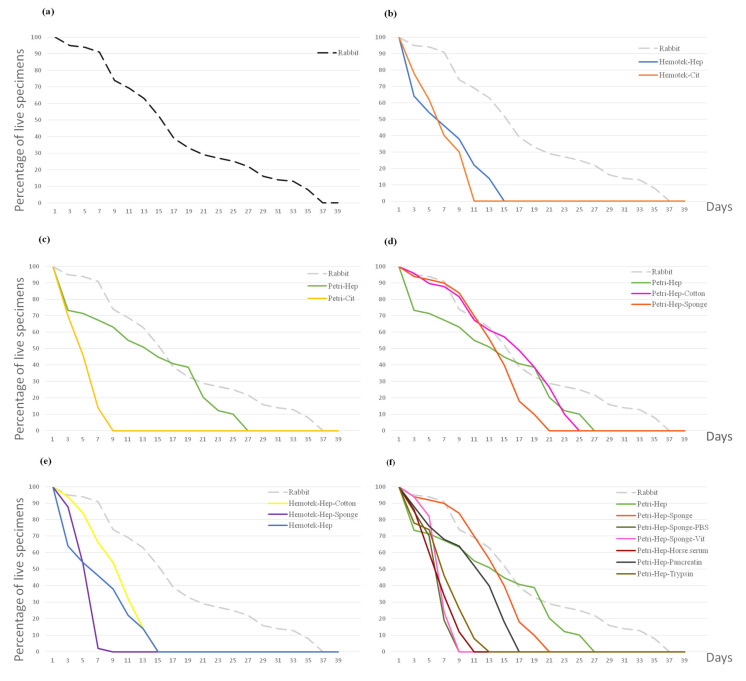
Curves representing the percentage of live lice during each feeding experiment (48 h). (**a**) Control feeding on a rabbit host. (**b**) Lice feeding on citrated and heparinised blood on a Hemotek system. (**c**) Lice feeding on citrated and heparinised blood on a Petri dish. (**d**) In vitro lice feeding on a Petri dish using a sponge and cotton as a supports for heparinised blood. (**e**) In vitro lice feeding on the Hemotek system using a sponge and cotton as supports for heparinised blood. (**f**) Blood supplements’ effects on lice longevity. Hep: heparin anticoagulant. Cit: citrate anticoagulant. Vit: vitamins.

We compared the effect of different blood anticoagulants on lice longevity. On both systems which were used, the Hemotek system and a Petri dish, the lice fed citrated blood showed lower survival rates. All lice were dead on the 9th and 7th days ([β = −0.28, *p* < 0.001 and β = −0.33, *p* < 0.001] for the Hemotek citrate and the Petri citrate vs. rab., respectively). In addition, when fed citrated blood, lice did not moult, and all specimens died at L1. In contrast, when fed heparinised blood, similar results were observed on the Hemotek system, with 50% mortality on the 5th day, and 32% developing to L2 on the 9th day post-hatching ([β = −0.26, *p* < 0.001] vs. rab.). When using the Petri dish with heparinised blood, moulting into L2 occurred on the 7th day for 56% of specimens and into L3 on the 15th day for 44%. Development to adulthood was observed in 20% of the lice 21 days post-hatching, but these females failed to oviposit and died five days later. Moulting into L3s and adulthood occurred four days later in this condition compared to the control group (Table 1). However, no significant differences were observed when compared to rearing on rabbits ([β = −0.11, *p*= 0.086] Petri–Heparin vs. rab.) (Figure 4b,c).

#### 3.2.2. Support

Different blood meal supports such as cotton and sponges were evaluated using both anticoagulants on both rearing methods. When lice were fed without any support, red blood cells sedimented and only the plasma remained in contact with the Parafilm membrane; therefore, only the plasma was taken as a blood meal. Moreover, without the supporting structure of cotton or a sponge, the Parafilm membrane surface was very smooth, limiting the specimens’ movements. When feeding on a Petri dish with heparinised human blood, no significant differences were observed between both supports. The sponge provided an irregular surface that facilitated the lices’ movement. Moulting into L2 was observed on day 7 for 90% of the specimens and into L3 on day 15 for 40%, with a statistically non-significant negative correlation observed when compared with the rabbit host ([β = −0.105, *p* = 0.094] vs. rab). Better results were obtained when using cotton as a blood meal support; moulting into L2, L3 and adulthood was observed on days 7, 15 and 21 for 88%, 56% and 26% of the specimens, respectively ([β = −0.05, *p* = 0.431] vs. rab.) (Figure 4d). For the Hemotek system, lice specimens that fed on heparinised blood through an imbibed sponge did not develop from the L1 stage, and all specimens died by day 9 ([β = −0.31, *p* < 0.001] vs. rab.). When using cotton as a blood meal support, 64% of the specimens successfully moulted into L2 on day 7; however, by day 15, no live specimens remained ([β = −0.21, *p* < 0.001] vs. rab.) (Table 1 and Figure 4e).

#### 3.2.3. Additions

We hypothesised that the blood meal was insufficient to maintain lice generations on the artificial membrane. Unfortunately, all additions tested drastically decreased lice longevity and were significantly different from the control group. When vitamins and PBS were introduced, all specimens died at L1 on day 9. Trypsin and horse serum also killed lice before they could moult. When pancreatin was added, all specimens moulted into L2 but died on day 17 (Table S1 and Figure 4f).

### 3.3. Blood Meal Inspection In Vivo

#### 3.3.1. Culture

For all tests that were carried out, the blood meal was discarded after each experiment. Multiple contamination controls were carried out, and no growth was observed on 5% sheep-blood-enriched Columbia agar (COS) (bioMérieux, Marcy l’Etoile, France) incubated at 37 °C, confirming the blood meal sterility. The blood meal support was also seeded on COS Petri dishes, and no contaminations were observed.

#### 3.3.2. Optical Microscopy

**Figure 5:**
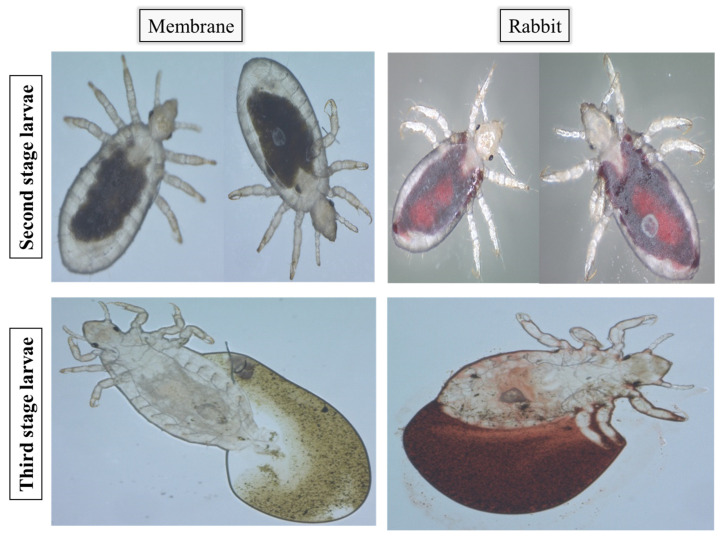
Images of the expulsed lice blood meal 24 h after feeding on artificial membrane and on rabbit host.

When evaluating differences in blood digestion 24 h after a blood meal, the lice and their expulsed blood meal clearly showed that the colour of the blood meal differed significantly between lice fed on a living host compared to those fed on an artificial membrane. Blood contamination, insufficient oxygenation or sulphur binding to haemoglobin may explain the greenish colour of the artificial blood meal (Figure 5).

### 3.4. Other Observations

The inspection of dead lice during every artificial meal enabled us to witness that the specimens died during the moulting process. In some cases, an abnormal moulting phenomenon was observed, with exuviae attached to the head but separated from the rest of the body. Moreover, the general activity of specimens fed on a membrane was lower than that of those fed on a live host.

### 3.5. Strategy Validation

In all previously tested conditions with an initial cohort of 50 lice, the females failed to oviposit. Therefore, we assumed that using a larger cohort might lead us to maintain successful rearing in vitro. The Petri dish was used to feed 500 newly hatched first instars on heparinised blood without any support or additions, this experiment was carried out in duplicate.

In the first trial, similar results to those using rabbit rearing were obtained [β = −0.043, *p* = 0.494]. On the 7th day post-hatching, L1 (1.39 mm) moulted into L2 (2.08 mm). Interestingly, moulting into L3 (2.36 mm) occurred for 13.8%, 4.1% and 64.6% of specimens on days 11, 13 and 15 and into adults for 10.8%, 3.6% and 43.2% on days 17, 19 and 21. Therefore, the maximum moulting percentage was observed on day 15 for L3, and 21 for adult females (2.68 mm) and males (3.1 mm), which is five days later than the control group. Females began ovipositioning on day 25 (with a four-day pre-ovipositional period) and a total of 52 eggs were produced. These were the best obtained results (BR).

**Figure 6:**
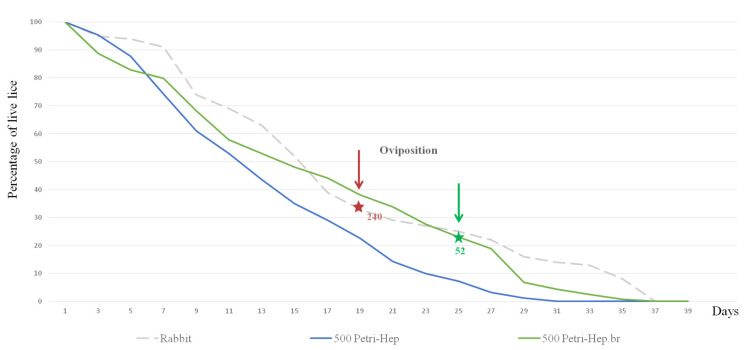
Percentage of live specimens fed on heparinised human blood using a Petri dish throughout time. Initial cohort for both trials is 500 newly hatched first-stage larvae. Hep: heparin anticoagulant; green curve: trial with the best results (BR).

For the second trial on 500 specimens, moulting into L2 was delayed by two days (day 9 post-hatching). Differentiation into adults took place for a small number of lice; therefore, the females failed to oviposit, and no significant differences were observed compared to those that fed on the rabbit. [β = −0.113, *p* = 0.7], and compared to the BR [β = −0.07, *p* = 0.25] (Table 1 and Table 2) (Figure 6). Artificial-membrane-developed lice assessed by SEM did not present any differences in their genitals or mouthparts when compared to the control reared on rabbits (Figure 2 and Figure 3) (Figure S1).

### 3.6. Cost

One rabbit can support six independent groups of lice; therefore, one experiment on a rabbit host is considered to be equal to one experiment on an artificial membrane with six feeding vessels. For instance, during each feeding, two injections of anaesthetising product are performed, a pot is discarded, and a new one is added to harvest the produced eggs. For the animal unit, each rabbit should rest for two weeks; hence, the rearing is initiated with six rabbits that can serve for no more than three years. In addition, cleaning the animal unit consumes more products than cleaning a lab bench. More importantly, because lice rearing on rabbits consumes more time, the technician’s working hours are longer, indicating higher remuneration. An important price difference was observed when comparing rearing in vivo and in vitro, and the cost of one rabbit-rearing experiment was EIR 28.88 and EUR 27.31 higher than using the Petri dish and Hemotek system, respectively (Table 3).

## 4. Discussion

In this study, we were able to compare the efficiency of several rearing methods. Indeed, rearing on a live rabbit host yielded better results, and no artificial feeding trials have yet allowed for a generational sequence. Using the Petri dish and heparinised blood, we were able to develop 33.8% of specimens into adults, after which 52 eggs were produced and 42.3% hatched. However, the 22 produced specimens failed to develop further than L1. In our initial trials, daily feeding provided better results than interval feeding (data not shown). It was shown that continuous feeding on human volunteers resulted in a higher egg rate production than interval feeding [27]. Nevertheless, the Or strain has adapted to interval feeding on rabbit; thus, we implemented the same feeding rate. Successful rearing is required to evaluate lice transmission capacity and to test ovicidal compounds in vivo. It is definitely more reliable to perform experiments on synchronised development stages of lice produced under controlled rearing conditions in the laboratory. In one recent study (2020), Pietri succeeded in producing fertile eggs using the Hemotek system [4]. However, in our situation, the Hemotek system performed significantly worse than the Petri dish system ([β = −0.22, *p* < 0.001] for Hemotek heparin vs. BR). We suppose that the metallic feeder might have affected the blood meal. Moreover, Pietri used rabbit blood as a blood meal for the Or body lice strain. This can explain the higher mortality rate in our trials, as we used human blood, and these lice have been feeding on rabbit blood since 1945. Another factor promoting the deaths of lice in our trials is the use of the Or strain. The authors used wild-collected lice for feeding on human blood in a polyprene tube cap. In this study, the low death rate and high egg production can be explained by the use of wild lice that are more resistant and adapted to harsher environments than the Or strain [26].

Before selecting the Parafilm, different membranes were tested. Quail and adult chicken skin were too thick for the lice to pierce. Pork intestines possessed the right thickness, but were not impermeable; therefore, the specimens were soaked in an oily substance. It was previously observed that newly hatched lice will not feed on a membrane thicker than 40 µm [20]. The artificial sebum used to attract lice turned the membrane greasy, preventing specimens from moving.

It was found that the anticoagulant had a significant impact on lice reproduction. EDTA decreased lice longevity, yet there was no difference between citrated and heparinised blood [21]. Our results contradict previous reports, and a significant difference between citrate and heparin on both rearing systems was observed ([β = −0.234, *p* < 0.001. β = −0.274. *p* < 0.001] for the Hemotek citrate and Petri citrate vs. BR, respectively). On citrated blood, all lice specimens died before reaching adulthood. This phenomenon may have been caused by the conservation of the blood for long periods of time, by blood dilution with the anticoagulant, since it is a liquid solution, or by citrate binding to calcium molecules and altering the composition of the blood [28]. Moreover, according to Lee et al., the dilution and quality of the blood meal may diminish fecundity [25]. However, heparin is lyophilised and prevents coagulation by neutralising some coagulation factors [29]. Mumcuoglu et al. claimed that lice did not feed on 27-week-old blood, and that when fed several blood types, lice took smaller meals [21]. Although the majority of our experiments were performed using two different blood groups, no significant differences in the feeding percentage was observed when lice were fed one or more blood types. In addition, Lee et al. previously stated that lice death synchronised with moulting [25]. We reached the same conclusion on this matter.

Lice inspection after feeding revealed a colour difference in the attributed meal. Blood was drawn from the donor’s vein and, therefore, the greenish blood colour is most likely due to lack of oxygenation. However, on live hosts, specimens pierce the skin and suck capillary blood, which is richer in oxygen. The rabbits’ shaved skin provided an irregular surface, facilitating lice movement. The temperature of the skin or membrane, in addition to the surface, affects the engorgement rate. Based on prior successful experiments reported in the bibliography, we assume that using fresh, defibrinated blood coupled with an agitation mechanism and the appropriate membrane thickness will theoretically be sufficient to maintain lice generations on an artificial membrane.

Respecting the welfare of animals in an institution is challenging. Cages, flooring and rabbits must constantly be kept clean. For each experiment, the technician must prepare the proper anaesthetic dose, and after weighing the rabbit, feeding can begin 15 to 20 min after injection and shaving. On a Petri dish, the entire preparation process takes about 15 min and requires less labour and time. Once the blood is heated, the lice begin feeding. We also compared the expenses of each technique and found that the use of a Hemotek device is slightly (EUR 1.57) more expensive than using a Petri dish. Hadenbank was able to maintain nine generations of human lice on a glass dish with a Parafilm membrane [30].

Further adjustments to the initial system are required to improve our results. First of all, daily feeding can lower the mortality rate of specimens. Second, occasional feedings on a live host can have a significant impact on lice fitness. Third, since the strain in question was fed on rabbits, it would be interesting to test rabbit blood as a blood meal. Finally, once a colony has been successfully reared, a cover incubator can be implemented in order to maintain a constant environmental condition.

Laboratory-reared body lice are extremely sensitive arthropods that cannot survive without frequent blood meals. Hence, some studies have used antibiotics to preserve the blood meal. Despite the fact that lice harbour an essential endosymbiotic bacterium, the use of antibiotics appears to have no effect on reproductivity and longevity [31]. Human lice rearing remains a challenging process for many laboratories, although it has been practiced on humans and animals since the early 1900s [32,33]. Successful lice rearing means abandoning the animal model and respecting animal welfare regulations. Without such trials, proving the capacity of lice to transmit disease, determining insecticide tolerance and assessing the lice life cycle will remain complicated.

## 5. Conclusions

The reported trials were insufficient to maintain multiple consequent lice generations on an artificial membrane. However, by comparing the different rearing conditions tested in this study, we identified the most appropriate and promising condition for maintaining one generation of human body lice on an entirely artificial system. The best condition was the Petri dish without any support, with heparinised blood, at 37 °C.

Moreover, the Petri dish system is significantly less expensive than in vivo rearing and slightly less expensive than the Hemotek system. Using sterile disposable material limits blood meal contamination and time consumption for material cleaning.

## Figures and Tables

**Table 1:** Number of live body lice, percentage in feeding and development stage on two rearing systems tested with different blood supports and blood types. Second and third trials: strategy validation on 500 specimens. Data for complementary trials are shown in Table S1.

System, Anticoagulant, Support	Blood Type/s	Days	1	3	5	7	9	11	13	15	17	19	21	23	25	27	29	31	33	35
Rabbit host		Live	100	95	94	91	74	69	63	52	39	33	29	27	25	22	16	14	13	8
		Exuviae	0	0	0	52	33	22	18	17	15	5	2	0	1	0	0	0	0	0
		Feeding %	98	95	96	97	97	92	96	92	92	84	82	85	88	90	87	85	76	70
Petri, Heparin	2 O+	Live	500	444	414	399	341	289	265	240	221	191	169	138	115	94	34	22	12	4
		Exuviae	0	0	0	15	250	40	11	155	24	7	73	30	15	0	0	0	0	0
		Feeding %	96	95	95	93	93	93	91	89	94	89	92	90	86	74	64	31	8	
Petri, Heparin	2 AB+	Live	500	477	439	371	305	265	218	175	145	113	71	50	36	16	6			
		Exuviae	0	0	0	0	27	126	46	34	47	8	2	5	1	0	0			
		Feeding %	95	95	98	98	98	94	92	95	94	82	72	78	62	50				
Petri, Heparin	2 O+, O−	Live	49	36	35	33	31	27	25	22	20	19	10	6	5					
		Exuviae	0	0	0	8	20	0	0	16	5	0	4	2	0					
		Feeding %	87	94	100	93	90	92	100	90	95	89	70	66	40					
Petri, Heparin, Cotton	2 O+, O−	Live	49	47	44	43	40	33	30	28	24	19	13	5						
		Exuviae	0	0	0	13	23	1	0	20	8	0	7	0						
		Feeding %	87	95	100	97	92	90	96	89	95	94	84	80						
Petri, Heparin, Sponge	O+	Live	50	47	46	45	42	35	28	20	9	5								
		Exuviae	0	0	0	10	22	0	0	16	3	0								
		Feeding %	96	95	95	95	91	89	85	77	90	60								
Hemotek, Heparin	O+	Live	50	32	27	23	19	11	7						First-stage larvae					
		Exuviae	0	0	0	0	14	7	0						Second-stage larvae					
		Feeding %	98	93	92	82	78	72	57						Third-stage larvae					
Petri, Citrate	A+	Live	50	35	23	7									Adult					
		Exuviae	0	0	0	0														
		Feeding %	98	88	78															
Hemotek, Citrate	A+	Live	50	39	31	20	15													
		Exuviae	0	0	0	0	0													
		Feeding %	61	80	55	66	60													

Highlight colour—blue: First-stage larvae, purple: Second-stage larvae, green: Third-stage larvae, yellow: Adult lice.

**Table 2:** Comparative development and fecundity of body lice stages fed through artificial membrane (in vitro) and live hosts (in vivo).

Condition	Petri Dish with Heparinised Blood Cohort: 500	Petri Dish with Heparinised Blood Cohort: 500	Petri Dish with Heparinised Blood Cohort: 50	Rabbit Host Cohort: 100
Engorgement	81%	86%	85%	89%
Longevity in days	35	29	25	35
First-stage larvae	6	8	6	6
Second-stage larvae	3	6	8	4
Third-stage larvae	6	6	6	6
Adult	19	9	5	19
No. of eggs/F	0.45	0	0	1.8
Egg hatch	43.6%	0	0	79%

No.: number. F: female.

**Table 3:** Cost for one feeding experiment on rabbit host, Petri dish and Hemotek system.

		Systems		
	Materials	Rabbit Host	Petri Dish (Six Vessels)	Hemotek System (Six Vessels)
Lice maintenance	Incubator ***	0.085	0.085	0.085
	Cloth *	0.32	0.32	0.32
	Pots *	0.23	0.23	0.23
Rearing	Heating pad **	0.107	NU	NU
	Shaving machine **	0.641	NU	NU
	Syringes *	0.1	NU	NU
	Anaesthetising product *	Rompun 3.462	NU	NU
		Imalgene 2.534	NU	NU
	Vacuum cleaner ***	0.04	NU	NU
	Rabbits **	6.41	NU	NU
	Cages ***	0.341	NU	NU
	Absorbent sheet *	2.4	NU	NU
	Water bath **	NU	0.213	0.213
	Heating plate **	NU	0.427	NU
	Hemotek system ***	NU	NU	1.068
	Petri dish *	NU	0.349	NU
	Blood *	NU	5.28	5.28
	Fridge ***	0.128	0.12	0.12
	Parafilm *	NU	0.11	0.057
Technician equipment	Technician salary	30.194	15.097	15.097
	Disposable lab gowns*	0.48	0.48	0.48
	Gloves *	0.08	0.08	0.08
	Lab coat *	0.18	0.18	0.18
	Hair net *	0.02	0.02	0.02
	Over socks *	0.12	NU	NU
	Technician training ***	0.512	NU	NU
Post-rearing	Floor and cage cleaning *	0.066	NU	NU
	Rabbit food *	2.721	NU	NU
	Rabbit maintenance *	0.705	NU	NU
	Surfasafe *	0.014	0.014	1.125
	Bleach *	NU	NU	0.22
Total		51.894€	23.007€	24.577€

*: one-time use. **: three years. ***: 15 years. NU: not used.

## Data Availability

Data are contained within the article and Supplementary Materials.

## Supplementary Materials

The following supporting information can be downloaded at: https://www.mdpi.com/article/10.3390/insects15030145/s1, Figure S1: SEM micrographs focusing on mouth ultra-structure from frontal view of all lice developmental stages after rearing them on Petri dish with heparinised blood compared to a rabbit host. Table S1: Complementary lice-rearing trials. Number of live body lice and percentage in feeding and development stage on two rearing systems tested with different blood supports, blood types and additives.
