# Supplementary material for: Development of a Technique Using Artificial Membrane for In Vitro Rearing of Body Lice Pediculus humanus humanus
Source: Insects. 2024 Feb 21;15(3):145. doi: 10.3390/insects15030145 (PMC10971218; doi:10.3390/insects15030145)
Supplement: Supplementary file 1 [file insects-15-00145-s001.zip › insects-2797612-supplementary.pdf]

Supplementary material

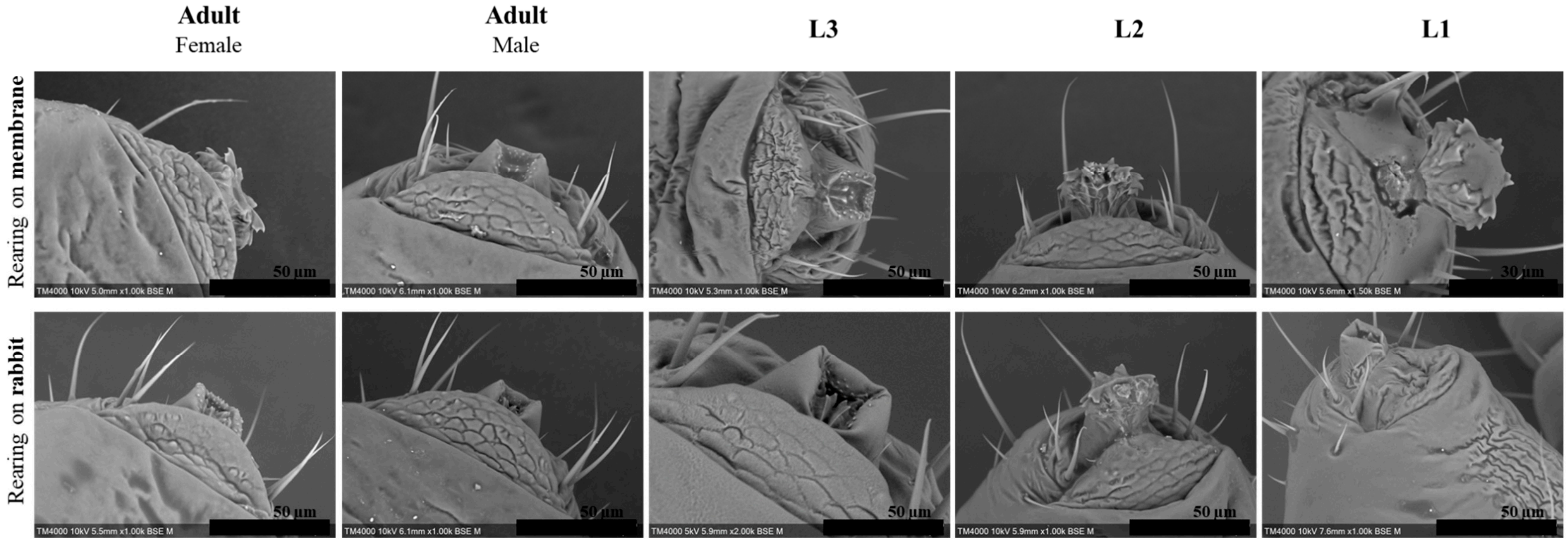

Figure S1: SEM micrographs focusing on the mouth ultra-structure from a frontal view of all lice developmental stages after rearing on Petri dish with heparinized blood, compared to rabbit host.

Table S1: Complementary lice rearing trials. Number of live body lice, percentage of feeding and development stage on two rearing systems tested with different blood supports, blood types, and additives.

System, Anticoagulant, Support, Additives	Blood type/s	Days	1	3	5	7	9	11	13	15
**Petri , Heparin , Trypsin 0,05%**	O+	Live	50	39	37	23	13	4		
		Exuviae	0	0	0	0	5	3		
		Feeding %	98	99	94	78	61	50		
**Petri , Heparin , Pancreatin 0,05%**	O+	Live	50	44	38	34	32	26	20	9
		Exuviae	0	0	0	4	23	2	0	3
		Feeding %	94	97	92	97	93	96	95	66
**Petri , Heparin , Horse Serum 20%**	O+	Live	50	43	30	17	6			
		Exuviae	0	0	0	0	0			
		Feeding %	84	91	88	90	90			
**Petri , Heparin , Sponge , PBS 25%**	2 O+ ,O-	Live	47	40	33	9				
		Exuviae	0	0	0	0				
		Feeding %	63	90	90	66				
**Petri , Heparin , Sponge , Vitamins**	O+ , O-	Live	50	47	41	12	First stage larvae			
		Exuviae	0	0	0	0	Second stage larvae			
		Feeding %	90	85	92	91	Third stage larvae			
**Petri , Citrate , Cotton**	A+	Live	50	29	19	0	Adult			
		Exuviae	0	0	0	0				
		Feeding %	92	84	82					
**Petri , Citrate , Spnge**	A+	Live	50	30	9	2				
		Exuviae	0	0	0	0				
		Feeding %	95	94	87					
**Hemotek , Heparin , Cotton**	O+ , O-	Live	50	47	42	33	27	16	7	
		Exuviae	0	0	0	0	18	5	0	
		Feeding %	98	97	95	90	77	81	42	
**Hemotek , Heparin , Sponge**	O+	Live	49	43	26	1				
		Exuviae	0	0	0	0				
		Feeding %	97	97	96	0				
**Hemotek , Citrate , Cotton**	A+	Live	50	54	29	25	26			

		Exuviae	0	0	0	0	0
		Feeding %	40	53	63	46	69
**Hemotek , Citrate , Sponge**	A+	Live	47	32	19		
		Exuviae	0	0	0		
		Feeding %	93	90	78		
